# Scattered migrating colony formation in the filamentous cyanobacterium, *Pseudanabaena* sp. NIES-4403

**DOI:** 10.1186/s12866-021-02183-5

**Published:** 2021-08-16

**Authors:** Hiroki Yamamoto, Yuki Fukasawa, Yu Shoji, Shumpei Hisamoto, Tomohiro Kikuchi, Atsuko Takamatsu, Hideo Iwasaki

**Affiliations:** Department of Electrical Engineering and Bioscience, Waseda University, Shinjuku, Tokyo, 162-8480 Japan

**Keywords:** Cyanobacteria, Colony pattern formation, Collective behavior, Cell motility

## Abstract

**Background:**

Bacteria have been reported to exhibit complicated morphological colony patterns on solid media, depending on intracellular, and extracellular factors such as motility, cell propagation, and cell-cell interaction. We isolated the filamentous cyanobacterium, *Pseudanabaena* sp. NIES-4403 (*Pseudanabaena*, hereafter), that forms scattered (discrete) migrating colonies on solid media. While the scattered colony pattern has been observed in some bacterial species, the mechanism underlying such a pattern still remains obscure.

**Results:**

We studied the morphology of *Pseudanabaena* migrating collectively and found that this species forms randomly scattered clusters varying in size and further consists of a mixture of comet-like wandering clusters and disk-like rotating clusters. Quantitative analysis of the formation of these wandering and rotating clusters showed that bacterial filaments tend to follow trajectories of previously migrating filaments at velocities that are dependent on filament length. Collisions between filaments occurred without crossing paths, which enhanced their nematic alignments, giving rise to bundle-like colonies. As cells increased and bundles aggregated, comet-like wandering clusters developed. The direction and velocity of the movement of cells in comet-like wandering clusters were highly coordinated. When the wandering clusters entered into a circular orbit, they turned into rotating clusters, maintaining a more stable location. Disk-like rotating clusters may rotate for days, and the speed of cells within a rotating cluster increases from the center to the outmost part of the cluster. Using a mathematical modeling with simplified assumption we reproduced some features of the scattered pattern including migrating clusters.

**Conclusion:**

Based on these observations, we propose that *Pseudanabaena* forms scattered migrating colonies that undergo a series of transitions involving several morphological patterns. A simplified model is able to reproduce some features of the observed migrating clusters.

**Supplementary Information:**

The online version contains supplementary material available at 10.1186/s12866-021-02183-5.

## Background

Bacterial colonies are formed through biological self-organization [1–5]. For example, pioneering studies on the morphology of *Bacillus subtilis* colonies demonstrated that colony morphology depends on the solidity and nutrient concentrations of the media [6, 7]. These results have been simulated numerically [1, 8].

Self-propelled bacteria often show complicated collective behaviors, such as the formation of dense moving clusters, which is exemplified by “wandering” (comet-like) and “rotating” colonies as described by Henriksen [9–11]. These types of clusters have not been rigorously defined. In the present report, however, we refer to migrating clusters consisting of high-density bacterial filaments that maintain their unity with a constant comet-like shape and move basically in a straight line while occasionally changing direction as (comet-like) wandering clusters. On the other hand, a moving cluster consisting of high-density filaments that maintains a similarly high degree of unity and continues to move in a rotational motion forming a circular orbit is called a (disk-like) rotating cluster. These colony patterns have been analyzed in detail, mainly in *Bacillus* and *Paenibacillus* species. For example, *Paenibacillus vortex* forms both wandering and rotating clusters when cells elongate in the presence of mitomycin C [12] or when they are co-cultivated with *Escherichia coli* [13]. *Paenibacillus Alvei* also forms wandering colonies [14], while *Paenibacillus* sp. NAIST15–1 forms both wandering and rotating colonies [4]. Meanwhile, *Myxococcus xanthus* is a bacterium that forms large moving clusters such as vortices, bundled circular patterns, side-by-side clusters, and rafts [15, 16]. In this species, EPS associated with the pilus [17, 18] and its trail [19] and its ability to reverse directions [16, 20] have been suggested to contribute to cluster formation via cell-cell interaction.

Motile cell aggregates have also been observed in cyanobacterial species which lack flagella, an appendage used in collective behaviors of *Bacillus* and *Paenibacillus* [21, 22]. Most intensively studied model would be the phototactic collective behavior in the unicellular species, *Synechocystis* sp. PCC 6803, which form finger-like migrating cluster toward light source [23–26]. Even in the absence of directed light illumination, some cyanobacterial species show complicated morphological patterns with motility. For example, *Phormidium* sp. KS shows spiral vortices on agar plates [27]. *Pseudanabaena* species isolated from a brackish mudflat in California was reported to show reticulate morphology in population during slurry aggregation [28]. More interestingly, another *Pseudanabaena* species (*Pseudanabaena geleata* OL-75-Ps) the comet-like wandering cluster is documented in a brief taxonomic catalog on the Bergey’s manual [29], while this report devoted only a few paragraphs regarding this issue; thus, detailed information is not available.

Here, we report the isolation of *Pseudanabaena* sp. NIES-4403, a filamentous cyanobacterium that forms both comet-like wandering aggregates and rotating clusters on solid media. At the macroscopic level, the mixture of wandering, and rotating clusters forms randomly scattered clusters that vary in size. The randomly scattered pattern on solid media looks similar to the “stellar” pattern that has been briefly described in *Paenibacillus Alvei* [30], while its mechanism of formation remains to be known. We initially considered the way of short-range interactions among neighboring filament (alignment), some positive feedback property to form aggregates, and transition between clusters to be important for the formation of scattered pattern. Based on this assumption, we investigated these clusters microscopically and performed quantitative analyses on the formation and motility of these clusters and on the temporal dynamics of the scattered pattern. In addition, mathematical modeling was also employed to reproduce the development of the scattered pattern.

## Results and discussion

### Scattered patterning of *Pseudanabaena* colonies

**Fig. 1:**
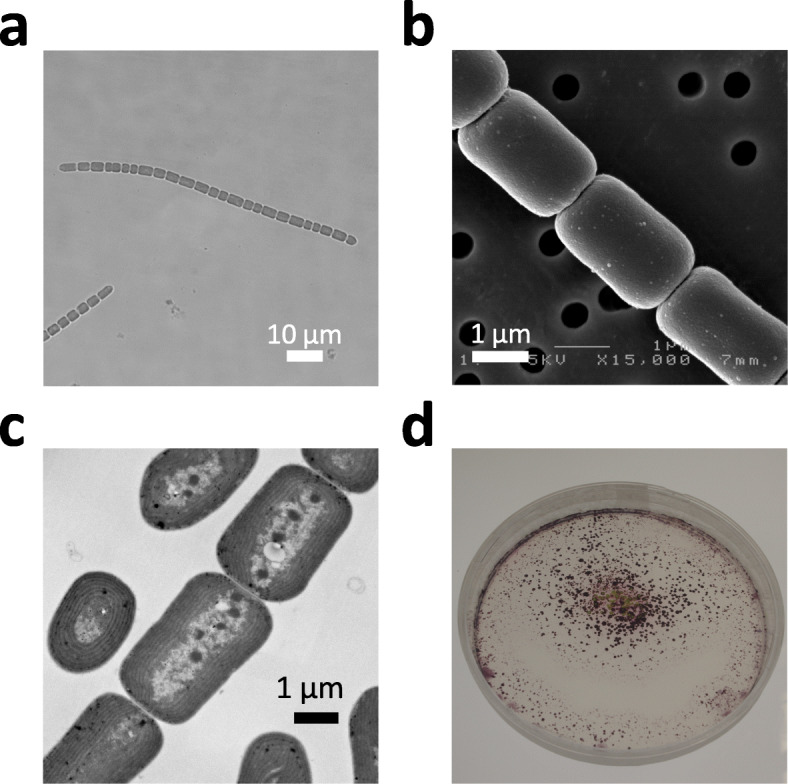
Morphology of *Pseudanabaena* sp. NIES-4403. **a**. Light microscopic image of cell morphology. **b**. Scanning electron microscope image of cell morphology. **c**. Transmission electron microscopy image. **d**. Colonies on solid BG-11 medium (90-mm plate) 12 days after inoculation at the center of the plate

**Fig. 2:**
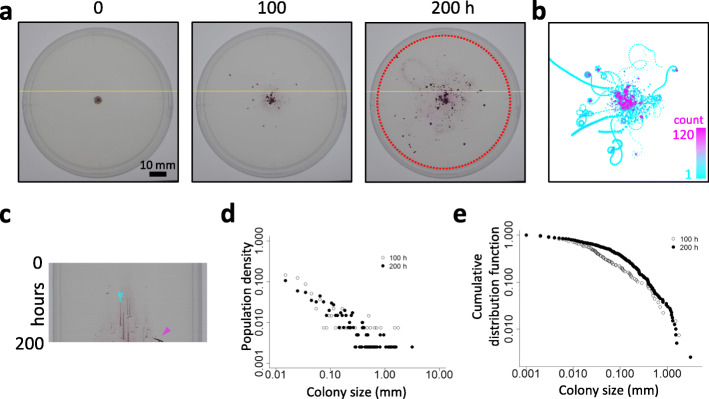
Scattered colony pattern of *Pseudanabaena* sp. NIES-4403 on solid BG-11 media. **a**. Time-lapse images of *Pseudanabaena* on a 90-mm plate. Hour 0 represents time that time-lapse photography started, corresponding to ~ 10 h after inoculating a cell suspension at the center of the plate. Time-lapse imaging was performed every hour, and the images were compiled into Movies S1. **b**. Trajectory of wandering colonies over 120 h from hour 0 of Movie S1. Color represents the passing count (log) (1 count, cyan; 120 count, magenta). **c**. A kymograph of colonies represented by yellow lines shown in panel **a** over a 200-h period (top, hour 0; bottom, hour 200). Cyan and magenta triangles represent a rotating disk and a comet-like wandering cluster, respectively. **d**. Colony size distribution inside the red dotted circle in panel **a** at hours 100 and 200 on a log-log plot. **e**. Cumulative distribution function of colony size at hours 100 and 200. It should be noted that the largest cluster located at the center is not considered because it is derived from the initial spot of inoculation; thus, its morphological pattern is due to both autonomous behavior and the artificial inoculation setting

We isolated a filamentous cyanobacterium (Fig. 1a–c) from a pond at Waseda University in Tokyo (35.706 oN, 139.708 oE). The cyanobacterium displayed remarkable colony morphology on solid media. Phylogenetic analysis of its16S rRNA gene has revealed that this strain is closely related to the genus *Pseudanabaena* (Fig. S1). Thus, we registered this strain as *Pseudanabaena* sp. NIES-4403 at the Microbial Culture Collection of the National Institute for Environmental Studies (NIES collection, Tsukuba, Japan). On BG-11 solid medium, this cyanobacterium was observed to develop randomly scattered clusters that varied in size (Fig. 1d). When a cell suspension was placed at the center of agar plates, growing cells glided on the surface and showed a series of dynamic collective behaviors (Fig. 2a, Movie S1). One collective behavior gives rise to (comet-like) wandering clusters, and these occasionally form vortices called (disk-like) rotating clusters. The rotating clusters may reach a diameter of ~ 1 mm (see below).

The randomly scattered pattern on solid media (Figs. 1 and 2d and a) looks similar to the “stellar” pattern that has been briefly described in *Paenibacillus Alvei* [30] (we prefer to call “starry-like”, though) and whose mechanism of formation remains unknown. Approximately 1 day after cells were inoculated at the center of the solid media (Fig. 2a left, hour 0 in Movie S1), highly dense, moving (comet-like) clusters started spreading and traveled around (Fig. 2a middle and right, hours 100 and 200 in Movie S1). Some of the clusters became organized into small circular orbits that sometimes coalesced when the head and tails of clusters attached, thus developing into a rotating disk-like cluster. Most of the rotating clusters kept rotating as the cells grew (Fig. 2b for passing count imaging), although a minor fraction of the cell population reverted back to comet-like clusters that travel throughout the surface of the medium (see below). A kymograph shown in Fig. 2c represents a time-dependent profile of bacterial cluster formation and its transitions. Transiently appearing patterns of dark dots and sloped short bars (magenta arrowhead) on the kymograph indicate the passage of comet-like wandering clusters, while vertical gradient lines (cyan arrowhead) represent rotating clusters that remain in one location and gradually expands in size. It should be noted in rotating clusters in *Bacillus* and *Paenibacillus* species gradually change their position [4, 9, 12], while that in *Pseudanabaena* kept their position for several days, that appeared as a long vertical line on the kymograph. Sudden end of this type of long vertical line may imply the transition from a rotating cluster to a wandering cluster.

### Random multiplication process implied in the scattered pattern formation

Interestingly, the probability distribution function (PDF) of cluster size distributions (including comet-like and disk-like clusters) follows a roughly straight line on a log-log plot, regardless of time after inoculation (hour 100 or 200 in Fig. 2d). This distribution is reminiscent of random multiplication processes that contribute to power-law or log-normal distributions and further underlie the behavior of complex systems in which various elements are connected to various stochastic factors from the past. Thus, the result in Fig. 2d suggests that the development and decay of bacterial clusters depend on a history-dependent random multiplication process. To better understand this process, the cumulative distribution function (CDF) of the colony size distribution is presented in Fig. 2e, and we tested whether this distribution is similar to either a power-law or log-normal distribution using a method described by Clauset et al. [31] implemented within the poweRlaw package [32] on R (for details, see Materials and Methods). They proposed that the null hypothesis H_0_ (the experimental data follows the power-law distribution) may be rejected if the *p-*value is less than 0.1. On the one hand, the *p-*values calculated to determine the degree of fit to the power-law distribution were 0.15 and 0.01 for the experimental data at hours 100 and 200, respectively. On the other hand, the corresponding *p-*values determining fit to the log-normal distribution ([31, 32] for details) were 0.12 and 0.89, respectively. We also performed the Vuong’s test, which is a likelihood ratio test for model selection using the Kullback-Leibler criterion [31, 32], to determine whether the model distribution is closer to power-law or log-normal. Here, the null hypothesis H_0_ (both distributions are equally far from the true distribution) is tested against the alternative hypothesis H_1_ (one of the test distributions is closer to the true distribution). A *p*-value greater than 0.1 indicates that it is difficult to determine which distribution is more appropriate, according to Clauset et al. [31]. According to our calculations, the *p*-values for the data at hours 100 and 200 are 1.02E-08 and 1.34E-03, respectively. Taken together, our results suggest that the power-law distribution is a better fit for the experimental data at hour 100, while the log-normal distribution is a better fit for the data at hour 200. This is consistent with our findings for the CDFs for data at hours 100 and 200, in which the former follows a straighter line on a log-log plot, which indicates a power-law distribution (Fig. 2e). This suggests that an additive fluctuation effect due to random multiplication processes is more apparent at hour 100, which is possibly due to rapid exponential growth of cells at this stage. Meanwhile, at hour 200, the effect of cell propagation is weakened due to slower growth.

### Comet-like wandering clusters

**Fig. 3:**
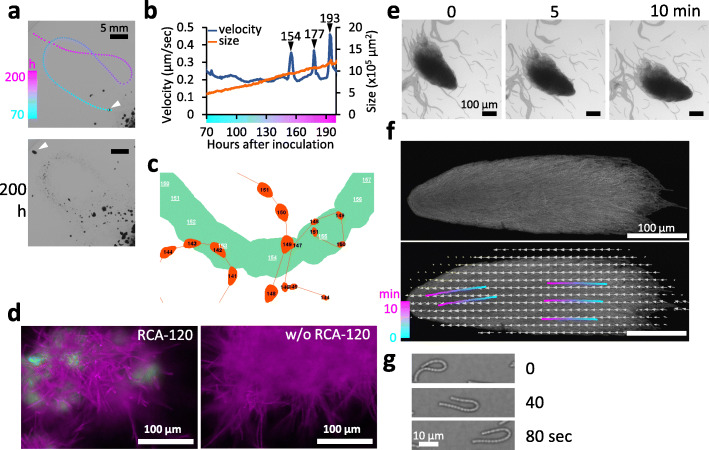
Comet-like wandering cluster displaying coordination in the direction of movement and velocity. **a**. Trajectory of a representative comet-like wandering cluster. Colors indicate the positions of the cluster in between hours 70 (cyan) and 200 (magenta). For more details, see Movie S2, which was extracted from Movie S1. **b**. Time course of changes in velocity and size of the cluster shown in panel **a**. Color bar on the horizontal axis is the same as shown in panel **a**. Arrowheads show the time when the velocity accelerated transiently. **c**. Trajectory of the same cluster during the first spike in velocity that peaked at hour 154 (Green). Orange represents trajectories of three other preceding clusters. It should be noted that the velocity of the cluster shown in cyan started to increase at hour 152 (panel **b**), when the cluster passed over a trail of a preceding cluster. From this time up to hour 156, the cluster passed over two other trails. **d**. Fluorescent microscopic images of RCA-120 stained cells. Magenta and cyan indicate autofluorescence of cells and stained EPS, respectively. **e. **Movement and morphology of a comet-like cluster wandering for 10 min. The video is provided as Movie S3. **f**. PIV analysis of the movement of the comet-like cluster with confocal images of autofluorescent cells. Color bars and arrows indicate trajectories of single cells within a period of 10 min (cyan to magenta) and the result of PIV analysis (velocity), respectively. The video is shown in Movie S4. **g**. U-shaped motion of a single filament

Figure 3a shows a representative trajectory during gliding movement of a comet-like wandering cluster on solid media for ~ 120 h (for movie, see Movie S2, which had been extracted from Movie S1). This cluster originated close to the central position (inoculation point) of the plate and then wandered along the route shown by the dotted lines (from cyan to magenta, Fig. 3a). During the course of traveling, the area occupied by the cluster almost doubled linearly (Fig. 3b, orange line). Clusters grow by (i) integration (unity) of multiple clusters and/or by (ii) cell growth. We then analyzed the growth of a cluster on Fig. 3a–b, wherein the size was determined to expand even when it wandered at peripheral positions of the plate where visible colonies were not present. This indicates that cells grow within wandering clusters. The cluster moved at a stable velocity of ~ 0.10–0.43 μm/s for most of the experiment (*n* = 55, Fig. S2, Table 1, for other detailed trajectories see also Fig. S3). However, on the trajectory shown in Fig. 3a transient increases in velocity were observed three times: at hours 154, 177, and 193 (Fig. 3b). Interestingly, at hours 177 and 193, the cluster was crossing over positions that it had previously passed. Thus, gliding speed may accelerate due to micro-environmental changes elicited by previously passing bacterial colonies. For example, a passing cluster may change the water environment on the solid surface by secreting mucilage. Notably, in *Paenibacillus* sp. NAIST15–1, an extracellular protein CmoA may play a role in wandering and rotating clusters by affecting water uptake from the agar medium [4]. The transient increase in cluster velocity at hour 154 did not occur at a location that it had previously passed; however, at least three other comet-like wandering clusters had previously passed this position (Fig. 3c). We suggest that the trails of these clusters affected the acceleration of the cluster we were monitoring due to the same reason that explains the accelerations at hours 178 and 195. In Fig. S3 we showed additional three trajectories of wandering clusters on another plate (Movie S3) for reproducibility. They also show essentially similar profiles: transient increase in the velocity corresponded with the timing of passing over trails (shown in arrowheads).

**Table 1:** Comparison of cluster velocities (μm/sec)

	Minimum	Maximum	Median	Average	Standard Deviation	Number
Rotating clusters	0.231	0.824	0.494	0.491	0.142	30
Wandering clusters	0.104	0.429	0.276	0.264	0.089	55

In gliding bacteria, secreted mucilage such as extracellular polysaccharides (EPS) are proposed to activate motility [20, 33, 34]. In *Oscillatoria*, *Mastigocladus* and *N. punctiforme* several studies have reported that trails with mucilage are stained with india ink [35–38]. Although we have repetitively tried this assay, we failed to obtain reproducible results. Instead, in some cases we observed trails exclude ink stain. Fig. S4a shows a comparison of the images taken just before and about 2 min after a drop of ink was applied onto the area where wandering colonies were observed. The trail area after the clusters have moved is much less stained with ink; in other words, ink has been eliminated from the trail area. By comparing the before and after images, it is clear that the white area is not the result of the elimination of the ink that was originally there after the movement of the cluster. This result indicates that the surface structure of the trail part is different from that of the basal solid media. Then, we tested if EPS is stained with either a lectin-based fluorescent reagent (fluorescein-conjugated RCA-120) or Alcian blue in *Pseudanabaena.* In hormogonia of *N. punctiforme*, RCA-120 stains EPS at each filament level [37], while we failed to observe it at this level. However, as shown in Fig. 3d and Fig. S4b, both RCA-120 and alcian blue staining resulted in positive signals around cell aggregates. Failure in positive detection of ink-stained trails or RCA-120 staining at single filament levels may be due too much higher solubility of EPS to trap ink particles or lesser amount of EPS secretion in *Pseudanabaena* compared with *N. punctiforme*. Though further studies should be followed for better understanding, taken together, these results indicate *Pseudanabaena* cells secretes EPS, which would contribute to change the surface structure of trails.

The stability of comet-like wandering clusters (for better resolution, see Fig. 3e and Movie S4) suggests a tight interaction among bacterial filaments. To determine the detailed architecture of a cluster, we observed its surface structure by confocal microscopic analysis (Fig. 3f, Movie S5). The cluster moved at a rate of approximately 100 μm within 590 s, without changing the relative positions of cells within the cluster, as indicated by particle image velocimetry (PIV) analysis (Fig. 3f). Some exceptions consisted of filaments near the head of the structure that did not bind tightly to the cluster. Matsuyama and Matsushita [39] reported that *Bacillus subtilis* has formed finger-shaped extending branches in which cells at the outermost tip wall remain immotile, while the inner cells randomly swirl, pushing the tip wall cells to extend the branch (division of labor). The tip wall of the comet-like wandering cluster in *Pseudanabaena* is also covered by layers of filaments that can be pushed by inner filaments that are aligned along the axis of the cluster. However, unlike *Bacillus subtilis*, the relative positions between the tip wall filaments and the inner nematically aligned filaments change little as the cluster moves (Movie S5). In *Pseudanabaena*, it is still unclear if the wall tip cell layer moves passively by being pushed by the following aligned filaments or by self-propelled, active motive force. It should be noted that even U- or horseshoe-shaped single filaments are able to move autonomously (Fig. 3g).

Cells on the bottom side of a cluster are stably attached to the solid agar surface, which means that the filaments in the upper layers, horizontally situated above the bottom side, do not directly associate with the agar surface. Therefore, another type of division of labor must be involved in wandering clusters in *Pseudanabaena*. In this case, the bottom side filaments appear to be more responsible for driving motility, while cells in the upper layers do not move. As the cluster becomes larger, it expands both two-dimensionally and along the Z-axis. Thus, if cells in the upper layers do not contribute to motility, then the speed of gliding movement should decrease. However, this slowing down was not observed. Therefore, we suggest that filaments in the upper layers also contribute somewhat to cluster motility. A simple possibility would be as follows: the filaments in the upper layers can move on top of those below and supply EPS which enhances the motility of the surrounding filaments. Thus, at any given time a subset of filaments from all layers could be at the leading edge of the cluster and enhance motility of the lagging filaments by EPS deposition.

### Disk-like rotating clusters

**Fig. 4:**
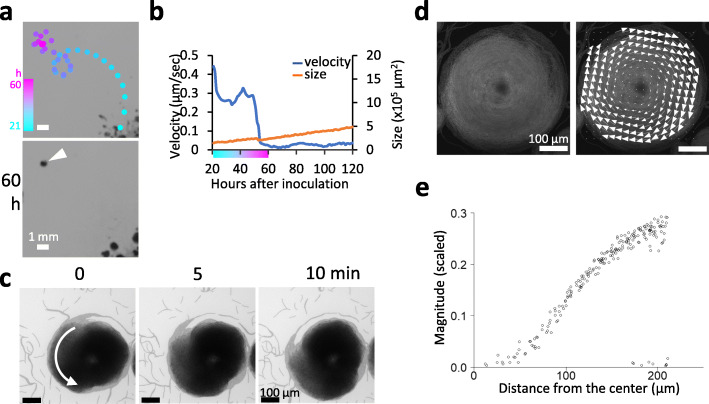
Rotating disk clusters. **a**. Trajectory of a cluster turning from a comet-like cluster to a rotating disk. Colors indicate the positions of the cluster at hours 21 (cyan) and 60 (magenta). Photographs show only the colony profile at hour 60, and the arrowhead indicates the resulting rotating disk. For more details, see Movie S6, which was extracted from Movie S1. **b**. Time course of changes in velocity and size of the cluster shown in panel **a**. Color bar on the horizontal axis is the same as shown in panel **a**. **c**. Movement and morphology of a rotating disk cluster within a period of 10 min. The video is provided in Movie S3. **d**. PIV analysis of the movement of the rotating disk cluster with confocal images of autofluorescent cells. Arrows indicate the result of PIV analysis (velocity). The video is provided in Movie S8. **e**. Correlation between the distance from the center of the rotating disk and the magnitude (velocity) of the PIV analysis. Detailed spatial distribution is shown in Fig. S6.

Figure 4a shows a representative trajectory of gliding movement for ~ 40 h on solid medium by a comet-like wandering cluster that was in the process of turning into a rotating cluster (see also Movie S6). The cluster originated from the center of the plate at hour 22 and then wandered along the route shown by the dotted lines (from cyan to magenta, Fig. 4a). Thereafter, the cluster transformed to form a compact circular orbit, resulting in a transition from the comet-like wandering colony to the rotating cluster at around hour 56. The size of the cluster decreased slightly during the transition into a disk-like rotating cluster (hour 56), which also occurred as a comet-like wandering cluster (Fig. 3b). However, the size was observed to increase linearly after hour 61 (Fig. 4b, red line). Although the disk-like rotating cluster appears to stay in place (Fig. 4b, blue line), this does not mean that cells in the disk-like rotating cluster do not move. As mentioned earlier briefly, at the cellular scale, filaments within a rotating cluster maintain a circular motion (Fig. 4c, Movie S4). Reproducibility of similar velocity and size progression profiles was confirmed for three other trajectories to show wandering to rotating mode transition on another culture plate (Fig. S5, Movie S7). We have observed that a large disk-like rotating cluster with a diameter of ~ 1 mm usually remains at a terminal collective mode; however, it maintains its rotation for more than 10 days. The vertical wavy lines on the kymograph shown in Fig. 2c indicate continuous, regular rotation. This is quite different from previously reported rotating clusters in *Paenibacillus* sp. NAIST15–1, which immediately stop rotating after forming large vortices [4]. Such collective aggregate movements also result in a scattered (discrete) colony pattern. In such vortices of bacteria, in general, cells at the edge of a rotating aggregate move faster than cells at the center [2, 40]. This was confirmed in *Pseudanabaena* by PIV analysis of a video of a rotating cluster that was recorded through a confocal microscope (Fig. 4d, Movie S8**,** and Fig. S6). Figure 4e shows distribution of velocities, which were measured from the center to the outermost part of the rotating cluster. The plots likely consist of three components: (1) a central zone measuring less than ~ 30 μm (Fig. 4e) where very slow or randomly moving filaments are dominant with lower filament density; (2) a rotating zone (~ 100 to ~ 180 μm in Fig. 4e) where the velocity is elevated depending on the distance from the center; and (3) a peripheral or outmost zone (~ 180 to ~ 220 μm in Fig. 4e) that contains a mixture of filaments that move at fast and slow speeds. If a rotating aggregate is perfectly rigid, the rotation speed *v(R)* = *ωR*, where *R* is the distance of the point from the center and *ω* is angular speed of the rotating cluster. Although the distribution of velocity shown in Fig. 4e is not perfectly on a straight line, the results confirmed the speed increased depending on the distance from the center. The sudden drop in the velocity at the peripheral/outmost zone can be attributed to the presence of immotile or slowly moving bundles or filaments being dissociated from the rotating cluster (see Movie S8). It should be noted that we cultured cells for confocal microscopy on 1.5% gelrite gels instead of agar for clarity (see Methods), while we used agar for observations. We confirmed that selection of gels did not affect much on cluster development and movement. Nevertheless, velocity of cluster migration can be slightly affected. Thus, velocity at the peripheral/outmost zone of rotating clusters (*n* = 30) was analyzed to be compared with that of migrating clusters on agar plates (Fig. S2 and Table 1). Interestingly, the velocity at the peripheral/outmost zone was found to be significantly faster than that of wandering clusters. Wandering clusters are subject to drag from the medium at their forefront, which would somewhat retard the movement. In contrast, at the periphery of the rotating cluster, which is aligned in a circular pattern, the filaments always follow the filaments in front. If EPS reduces friction, any cells at the periphery of the cluster will benefit from it, and as a result will likely tend to be faster than wandering clusters.

As for the direction of rotation of the disk-like clusters, both clockwise and counterclockwise seem to appear in almost the same way, as judged from the side of the top (bacterial) surface. When we examined the orientation of 10 random rotating clusters on each of three plates, the ratio of counterclockwise to clockwise was 6:4, 7:3, and 4:6, respectively, for a total of 56.7% clockwise and 43.3% counterclockwise. Even when comparing the two neighboring rotating clusters, some pairs rotated in the same direction, while the other pairs were in different directions. Basically, the rotational direction seems not change from the beginning of the disk-like rotating cluster formation. Change in the direction was only exceptionally observed when the rotating cluster was subjected to large perturbation by merging of another wandering cluster. Therefore, the direction of rotation is primarily defined by the bending direction of a wandering cluster when it bends into a circular trajectory for the formation of a rotating clusters. The direction in which the wandering cluster bends seems not particularly directional, and is considered to be primarily random.

### Bundles and single filaments

**Fig. 5:**
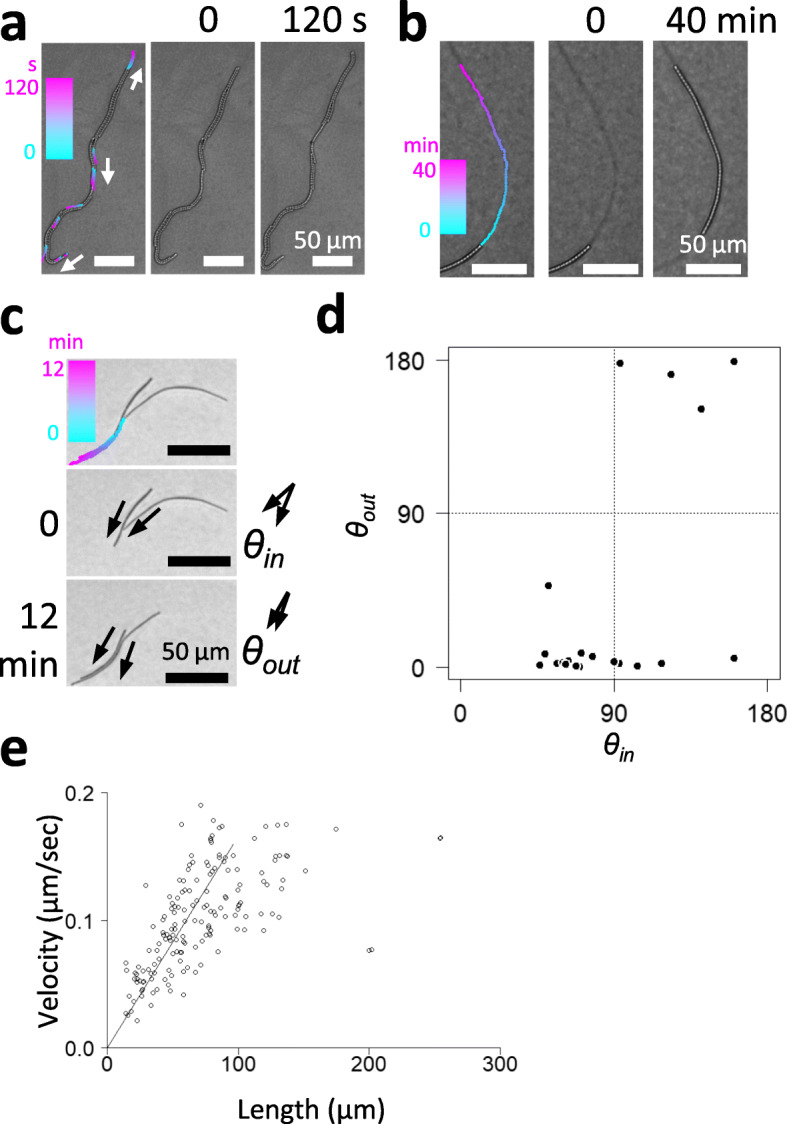
Trail following and nematic alignment (bundle formation) of filaments. **a**. Microscopic view of bundled filaments. Colors indicate the trajectory of the tip positions of each filament (0 s in cyan to 120 s in magenta). The video is provided in Movie S9. **b**. A filament following a trail shown as a thin line before the filament. Points indicate positions of the tip at each time point shown by color (0 min in cyan, 40 min in magenta). The video is provided in Movie S10. **c**. Time-lapse images of colliding filaments. Points indicate the trajectory of tip positions shown by color (0 min in cyan, 12 min in magenta). The video is provided in Movie S11. **d**. Distribution of incoming angle and outgoing angle between colliding filaments. **e.**  Relationship between the velocity and length of single filaments. Line indicates the result of regression analysis performed on data with values less than 95.98 μm (for details, see text and Fig. S7)

Except for the comet-like wandering clusters and rotating clusters, most bacterial filaments move alone or form bundles (Fig. 5a). A bundle is formed when several filaments align themselves along their longitudinal axis. In comet-like wandering clusters and disk-like rotating clusters, most of the filaments move in coordination with other nearby filaments. By contrast, filaments in bundles are not always stably aligned, and bundles may often dissolve or merge with each other. For example, Fig. 5a shows most of the filaments in the bundle move to the lower side; however, two filaments of the bundle moved to the tip of the bundle at the upper side, resulting in the division of this bundle into three bundles (Movie S9). As mentioned above, some filaments may move against the lateral axis to form a U-shape (Fig. 3g). In other filaments moving along their longitudinal axis, some may switch or “reverse” their direction of movement. We tended to observe such reversals in movement with dispersed filaments or with relatively free moving filaments at the peripheral region of cellular aggregates, and we rarely observed the phenomenon inside of high-density clusters. As filaments move, they leave behind a kind of trail that other filaments may follow, as if being guided (Fig. 5b, Movie S10). Although the details of such trails remain unclear, it is likely that the mechanism depends on either a groove created on the solid surface or on secreted EPS [20, 34] or both. In any case, the ability to follow trails should facilitate collective behavior [19]. As mentioned above, *Pseudanabaena* cells secrete EPS, although more detailed analysis is necessary to determine if it is involved in trail formation. When two filaments collide, in most cases, the filaments do not cross paths, but instead align with each other to form a bundle (Fig. 5c, Movie S11). Figure 5d shows the quantitative distribution of the cell alignment or the anti-alignment as a function of incoming angle. Plots shown on the bottom line (*θout*≃0) and on the top line (*θout*≃90) indicates that colliding filaments resulted in parallel alignment and antiparallel alignment, respectively. This characteristic, also known as “nematic alignment,” has been observed in *Myxococcus xanthus* [41], and this appears to be a key collective behavior in *Pseudanabaena* as well. It should be noted that in high-density clusters, neighboring filaments are essentially aligned with each other.

The velocity of a single filament gliding on a solid surface without following trails (i.e., moving on a virgin field) ranged from 0.02 to 0.19 μm/s, depending on the filament length (Fig. 5e). Below a length of 200 μm, filament velocity appears to be proportional to the length (Fig.5e). The correlation is higher at lengths below ~ 100 μm (Fig. S7), which produces a Spearman’s rank correlation coefficient of 0.74. The length-velocity correlation has also been reported in *Phormidium* sp. [27], which is another cyanobacterium whose average velocity increases with filament length, which the authors ascribed to the lowering occurrence of the reversal behavior (in that report, higher velocity represents longer net displacement). By contrast, Fig. 5e, showing the relationship between velocity and length of filaments, is based on the velocity of filaments that did not reverse their direction of movement during observation. We were aware that the reversal behavior is more frequently observed for shorter filaments, especially during early development immediately following inoculation. It is possible that each cell has motors that are more effective in longer filaments. In addition, or alternatively, cellular motors may be better synchronized in the longer filaments (up to ~ 200 μm). Moreover, it is also possible that longer filaments secrete more mucilage, which can reduce friction between the filament and the solid surface. It should be noted that the comet-like wandering colony consists of thousands of filaments, and it moves at a velocity that is comparable or slightly higher than the maximal speed of single filaments (0.19 μm/s). This velocity may increase transiently (up to 0.5 μm/s) when the colony crosses over pre-existing trails (Fig. 3b, c, and S3). Thus, even though the nematic alignment of thousands of filaments may be expected to facilitate movement and increase velocity to some extent, it likely contributes more to unifying the direction of motion and the speed of each filament.

The mechanism of gliding motility in *Pseudanabaena* remains unknown. However, several lines of studies have strongly suggested that gliding of filamentous cyanobacteria is driven by a polysaccharide secretion system known as the junctional pore complex (JPC). In differentiated motile hormogonia in *Nostoc punctiforme,* JPC is formed with arrayed ring structure of type IV-pilus-like systems, which are encoded in part by *pil* and *hps* genes [37, 42–44]. The *pil* and *hps* genes are conserved in essentially all filamentous cyanobacteria, as are several other genes that are important for motility in *N. punctiforme*, including those coding for the *hmp* chemotaxis-like system[37, 43, 44] . Indeed, the *pil* and *hps* genes are reported to be conserved in another *Pseudanabaena* strain, sp. PCC 7367 [43]. In *N. punctiforme* appearance of dense rafts with nematic alignment is often observed, while they do not typically separate from the inoculum site [44]. In addition, wandering masses is exceptionally observed in the *hmpW* mutant [45] under phototaxis conditions. The arrayed ring-based JPC has also been observed in *Phormidium* species which forms vortex clusters [27, 36]. Thus, we suggest high-density cluster formation in *Pseudanabaena* is also based on similar gliding mechanism. Nevertheless, to explain scattered high density cluster patterns exclusively observed in *Pseudanabaena*, further understanding on additional aspects like cell-cell interactions and physical property of filaments must be necessary.

### Transition of colony patterns

**Fig. 6:**
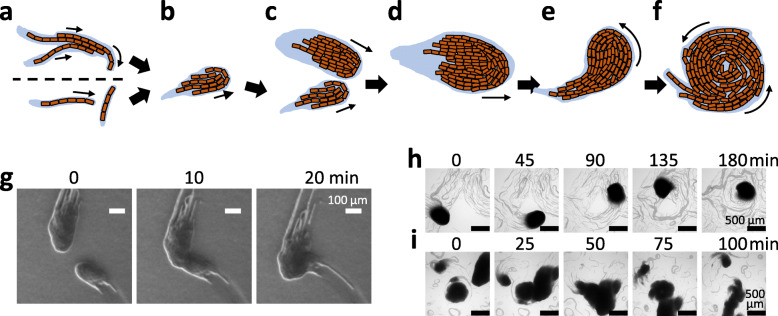
Development of comet and disk clusters from a single or a bundle of filaments. **a-f**. Schematic representation of the transition of colony patterns. Bacterial filaments and possibly secreted mucilage are shown in brown and pale blue, respectively. Spontaneous bending of a protruding filament in a bundle (**a**, upper) or collision of two single filaments crossing paths (**a**, bottom) leads to the formation of a “top covered” bundle (**b**) which is a precursor to a comet-like wandering cluster. Collision of comets (**c**) and propagation of cells enlarge the size of comet-like clusters (**d**). Change in the direction of the movement (**e**) can spontaneously lead to a self-following orbit, which develops into a rotating disk (**f**). Detachment of filaments and collision-based decay of the clusters also occur, leading to transitions from larger clusters to smaller clusters, bundle, or filaments. **g-i**. Time-lapse images of transitioning *Pseudanabaena* cluster patterns. **g**. Enlargement of comet-like cluster by collision of two comets. The video is provided in Movie S12. **h**. Transition from come-like wandering cluster to self-following orbit, developing into a rotating disk. The video is provided in Movie S13. **i**. Collision of a comet-like cluster (moving from the upper right to the center) to a rotating disk (center), leading to the collapse of the disk and reversion to a comet-like cluster. The video is provided in Movie S14

During the course of cultivation, clusters may change their patterns to any of those previously described (bundle, comet-like wandering cluster, or disk-like rotating cluster). When the density of cells/filaments is determined to be relatively low (early after inoculation), single filaments may collide, and align into a bundle as mentioned above. Figure 6a–f present a schematic diagram of the possible steps in the development of clusters. We suggest that comet-like wandering colonies develop from top covered bundles, as shown in Fig. 6a and b. Figure 6a (upper panel) shows a protruding filament at the outer side of a bundle as it spontaneously changes its curvature, while other neighboring bundles move forward, thus pushing the curved filament to form the top cover of the comet-like precursor. The lower panel of Fig. 6a shows that a filament or bundle may collide against a filament perpendicular to the direction of the former, thus forming a comet-like precursor. This top cover plays an important role in keeping the alignment of filaments stable, enabling them to move uniformly as an organized wandering cluster, at least at the initial stage. Otherwise, filaments easily dissociate from each other as it is often observed for bundles (i.e., “bundle to single” transition). The top covering filament at the initial stage also works as a trap, collecting bundles, or single filaments that enable the comet-like precursor to grow.

We propose that the formation of a high-density cluster requires a positive feedback system in order to facilitate aggregation. Comet-like wandering colonies become larger (Fig. 6b–d) via both cell propagation and collision-based uptake of bundles or other comet-like wandering clusters, as shown in Fig. 6g and Movie S12. Well-developed, comet-like wandering clusters may spontaneously curve due to local differences in velocities of filaments inside the cluster, possibly triggered by friction from the solid surface of agar, or partial attachment with trails (Fig. 6e). As a comet-like wandering cluster enters into a circular orbit, it turns into a disk-like rotating cluster (Fig. 6h, Movie S13). As mentioned earlier, most of such disk-like clusters maintain a stable rotation, thus developing into a terminal cluster. Nevertheless, some disk-like rotating clusters may revert back to comet-like wandering clusters by colliding with another comet-like wandering cluster as shown in Fig. 6i and Movie S14. On the kymograph shown in Fig. 2c, this type of transition is represented by the sudden termination of vertical lines (rotating disk-like clusters).

### Mathematical modeling of the scattered pattern

As shown above, *Pseudanabaena* filaments tend to move with nematic alignment (Fig. 5c and d), and maintain a certain degree of curvature in its movement. These properties are not limited to bacteria, but are also observed in the collective movements of microtubule in vitro and in the collective behavior of *Caenorhabditis elegans*, where the emergent patterns vary in morphology from vortical in the former to reticular in the latter. Sumino et al. [46] modified the classical Vicsek model [47] to propose a simple model which explains the vortices formation of microtubules. Sugi et al. [48] then showed it is also applicable to the reticular morphology in *Caenorhabditis elegans* population. Thus, this model is a highly general one to reproduce some important features of different morphology depending on parameter set with small number of variables based on the following two rules: (i) the direction of movement of each particle attains uniformity by nematic order; and (ii) the rotation rate of particles is maintained for a relatively long period. We wanted to ask to what extent we could reproduce the scattered pattern of *Pseudanabaena* with this model with some modifications. It is important to note that in this study, we are trying to reproduce at somewhat over-simplified level whether scattered colonies, comet-like wandering colonies, and rotating colonies can occur even when based on minimalist assumptions. The previous model applied for *C. elegans* was run in a torus field to reproduce a small space, and resulted in the formation of a dynamical network and the stabilization of particle movements. By contrast, we ran our modified model in infinite space with locally condensed particles at the initial position to represent the actual inoculation situation (Fig. 2a). Under this modified condition, we predict that particles will not form a stable pattern and, in the end, will diffuse into infinite space. Details of the modified model are expressed as:

1 $$ {\mathrm{r}}_{i,t+1}={\mathrm{r}}_{i,t}+{v}_0{\mathrm{e}}_{\theta_{i,t+1}}+{v}_0\sum \limits_{r_{ij}<{r}^r}{\mathrm{F}}_{ij}^r+\frac{1}{N_i}{v}_0\sum \limits_{r^r<{r}_{ij}<l}{\mathrm{F}}_{ij}^a $$

2 $$ {\theta}_{i,t+1}={\theta}_{i,t}+{\omega}_{i,t+1}+\frac{1}{N_i}\sum \limits_{r^r<{r}_{ij}<l}\mathit{\sin}2\left({\theta}_{j,t}-{\theta}_{i,t}\right) $$

3 $$ {\omega}_{i,t+1}={\omega}_{i,t}-\frac{\omega_{i,t}-{\omega}_0}{\tau }+\sqrt{\frac{2}{\tau }}{\sigma}_{\omega }{\xi}_i $$

4 $$ {\mathrm{F}}_{ij}^r={k}^r\left({r}_{ij}-{r}^r\right){\mathrm{e}}_{ij} $$

5 $$ {\mathrm{F}}_{ij}^a=\frac{k^a}{r_{ij}}{\mathrm{e}}_{ij}, $$

where **r**_*i*, *t*_ is the position of a particle *i* at step *t*. The particle *i* then moves to ${v}_0{\mathbf{e}}_{\theta_{i,t}}$ at step *t*, where *v*_0_ and *θ*_*i*, *t*_ are the unit velocity and the direction of motion, respectively. ${\mathbf{e}}_{\theta_{i,t}}$ is the unit vector in the direction of *θ*_*i*, *t*_, The direction is derived from rotation rate *ω*_*i*, *t*_ and that of other *N*_*i*_ particles in *r*^*r*^ < *r* < *l* as an effect of alignment. *l* is the distance of interaction or filament length. *ξi* is normal random number with means of 0 and standard deviation of 1. The particle also receives attractive force ${\mathbf{F}}_{ij}^a$ (*r*^*r*^ < *r* < *l*) or repulsive force ${\mathrm{F}}_{ij}^r$ (*r* < *r*^*r*^) from neighbor particles. Parameters used in our simulation are listed in Table 2.

**Fig. 7:**
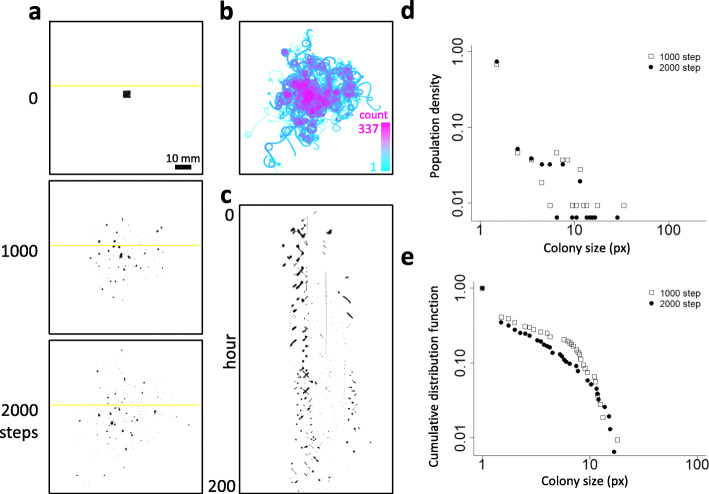
A simple self-propelled particle model reproducing the scattered colony pattern. The organization of the panels in this figure (simulations) is the same as that of Fig. 2 (experimental data). **a**. Simulation of scattered (discrete) colony pattern formation. For details, see Movie S. **b**. Trajectory of wandering agents over 2000 steps from time 0 of Movie S15. Colors represent the passing count (log) (1 count, cyan; 337 counts, magenta). **c**. A kymograph of agents on yellow lines in panel **a** over 2000 steps (top, step 1; bottom, step 2000). **d**. Colony size distribution of the model at steps 1000 and 2000 of Movie S15. **e**. Cumulative distribution function of the aggregation size of the model at steps 1000 and 2000

These parameters are essentially the same as those reported in a previous model [48], except the attraction term in Eq. (1) is divided by particle number *N*_*i*_ in order to avoid excessive increments of attraction under high-density conditions. We employed the newly fitted parameters as summarized in Table 2 for our simulation, the results of which are presented in Fig. 7 and Movie S15, which correspond to the in vivo experimental observations shown in Fig. 2 and Movie S1, respectively. In our simulation, particles have gathered and moved collectively like a comet-like wandering cluster (Fig. 7a and b, Movie S16) that the kymograph on Fig. 7c represents short, sloped bars. Some of these clusters entered into circular orbits as represented by periodically appearing short, sloped bars aligned to the vertical axis of the kymograph (Fig. 7c). The circular orbits of the simulated clusters were found to be transient (Fig. 7c and Movie S17), similar to comet-like wandering clusters moving tentatively on circular trails. It should be noted that this simple simulation does not implement the effects of mucilage secretion to facilitate trail-following and cell propagation, both of which contribute to change the density and size of aggregates. Nevertheless, the scattered colony patterning in *Pseudanabaena* (Fig. 2a) is at least partially reproduced by our model (Fig. 7a) with somewhat similar trajectory profiles (Figs. 2b and 7b). Colony size distribution and the cumulative distribution profiles observed in our experiments (Fig. 2d and e) were also reproduced in our simulation (Fig. 7d and e), to some extent. We tested whether the cluster sizes generated from empirical data would follow either a power-law or log-normal distribution (Fig. 2e) but failed to show which is more appropriate; that is, the *p-*values of the Vuong’s likelihood tests compare our results to the power-law and log-normal distributions were 0.87 and 0.15 at steps 1000 and 2000, respectively. Nevertheless, our results are consistent with the assumption that a random multiplication process contributes to the formation of scattered colony patterns in *Pseudanabaena*.

**Table 2:** Parameters and values of the model

Term Value	Description
*v*_0 _0.1	Mean velocity
*l * 1.0	Length of filament
*r*^*r *^0.2	Range of repulsive force
*ω*_0 _0.0005	Mean rotation rate
*σ*_*ω *_0.2	Standard deviation of rotation rate
*τ *500	Correlation time of rotation rate
*k*^*r *^10	Coefficient of repulsive force
*k*^*a *^0.1	Coefficient of attractive force

Although the model reproduced some features of the scattered pattern with migrating clusters, In the present simulation, all the parameters are treated as dimensionless and simulated in infinite space in order to qualitatively reproduce the spatial pattern. Thus, it lacks many variables which must be included in the bacterial collective system. For example, clusters in the simulation shown in Movie S16 tend to be much easily collapsed compared with that in bacterial population shown in Movie S1 and S2. It suggests that the nematic alignment among neighboring filaments is not sufficient to maintain the long-lasting unification of the migrating clusters. To compensate for this deficiency in the present simulation, incorporating a change in the surface state of the medium due to mucilage and the effect of cellular propagation and cell length elongation would be important. Since EPS-induced changes in the surface condition of the medium may increase in the ease of filament passage and the speed of wandering clusters (Fig. 3b, 3c and Fig. S3), it may also affect the integration of the population. For reproducing the morphological profiles observed in *Pseudanabaena*, incorporation of many parameters must be considered, such as the velocities of the filaments and clusters, filament length, more detailed interaction among filaments, the effect of EPS, the effect of drag and frictions and cell propagation.

## Conclusion

We describe the morphology of colony pattern formation based on the collective behaviors of the filamentous cyanobacterium, *Pseudanabaena* sp. NIES-4403. Aggregates were determined to develop into randomly scattered clusters varying in size and further consist of a mixture of comet-like wandering clusters and disk-like rotating clusters. Our study suggests that the following processes are key to pattern formation: (1) trail following of filaments possibly through polysaccharide secretion and/or groove formation on the solid surface, (2) bundle formation with nematic alignments, (3) top covering-induced formation of comet-like wandering clusters, (4) formation of disk-like rotating clusters through spontaneous self-following of the wandering cluster, and (5) collision-based transition among different cluster types. Based on the simple assumptions of nematic interaction and temporal maintenance of the direction of motion, our agent-based model reproduced some characteristics of the scattered colony formation. Although the observed morphologies were observed under artificial conditions, we speculate that these features contribute to the adaptive fitness of this species in its natural environment. For example, the formation of a highly dense cluster without long-distance migration, as exemplified by the disk-like rotating cluster, should be of advantage in forming a stable biofilm under preferred growth conditions. In addition, the ability to coordinate migration via a comet-like wandering cluster moving at maximal speed should facilitate colony expansion or escape from undesirable condition. In other words, this cyanobacterial species has developed its own strategy to form biofilms that include some positive feedback-based aggregation and dispersing processes. The morphological patterns produced under pure culture conditions on an artificially flat solid surface are manifestations of a strategy that has evolved under natural conditions. Needless to say, natural environment is much more complicated and thus formation and characteristics of migrating clusters must be influenced by external stimuli such as light and chemoattractors. It should be noted that at the beginning of isolation of the strain in 2007 the strain had shown positive phototaxis toward the light source, while during successive inoculation at the lab it turned to show weak negative phototaxis (Fig. S8), most likely due to spontaneous mutation. Even under this condition, formation of both wandering and rotating clusters are evidently observed. The shape of photo-avoiding clusters looked similar to comet-like clusters (Fig. S8). Under photo-avoiding conditions, the variation in the wandering direction would be reduced, increasing in the directness opposite to the light. Further detailed quantitative and morphological analysis should be conducted to compare collective behaviors under more natural conditions and under simple experimental conditions.

## Methods

### Strains and culture

*Pseudanabaena* sp. NIES-4403 was isolated on BG-11 solid medium by H.I. from pondwater in the Nishiwaseda Campus of Waseda University, Tokyo, Japan (35.706 oN, 139.708 oE). Cells were cultured on BG-11 medium [49] containing 1.5% of Bacto agar (BD Falcon, USA) under continuous light illuminated by fluorescent lamps (around 30 μmol m^− 2^ s^− 1^) at 30 °C. Subculturing was performed every 2 weeks by inoculating 4 μl of cell suspension on fresh BG-11 agar plates.

### Phylogenetic tree analysis

A region of the 16S rRNA gene was then amplified by colony PCR using primers 27F (5′-AGAGTTTGATCCTGGCTCAG-3′) and 1494R (5′-GTACGGCTACCTTGTTACGAC-3′). The amplified DNA fragment was further cloned into the pGEM-T Easy vector (Promega, USA) and transformed into *E. coli* JM109 cells (Takara Bio, Japan). The PCR-derived segments of the resulting plasmids were sequenced (Applied Biosystems 3730xl, Thermo Fisher, USA) using the same primers, and the resulting sequence was compared to 16S rRNA gene sequences from 29 cyanobacterial species (downloaded from the Ribosomal Database Project (RDP) on Mar 23, 2018) [50]. Twenty-four sequences with higher BLASTn (version 2.2.28+) similarity scores [51], excluding the sequences of “Uncultured Bacterium” and 5 sequences of representative cyanobacterial species (*Synechocystis* sp. PCC 6803, *Nostoc punctiforme* PCC 73102, *Anabaena* sp. PCC 7120, *Anabaena variabilis* ATCC 29413, and *Synechococcus elongatus* PCC 7942) were chosen to compare with the sequence derived from *Pseudanabaena*. These 16S rRNA gene sequences and that of *Pseudanabaena* sp. were aligned with MUSCLE (version 3.8.31) [52], calculated with ClustalW (version 2.1) [53], and used to generate a phylogenetic tree using Archaeopteryx (version 0.972 9 m) [54] (Fig. S1).

### Imaging of colony patterns

Microscopic images were taken using a CCD camera (RETIGA EXi FAST 1394; QImaging, Canada) coupled to an inverted microscope (IX71, Olympus, Japan) equipped with UPlanFLN (4x) and LUCPlanFLN (20x) objectives (both Olympus). The system was controlled by SlideBook software (Intelligent Imaging Innovations, USA). Confocal microscopic images were taken using the FV-1000D system (Olympus) connected to an automated microscope (IX81, Olympus) equipped with the LUCPlanFLN (20x) objective (Olympus) and controlled by FV10-ASW software. Five hundred fifty-nine nanometre laser excitation was used to visualize the autofluorescence from cyanobacterial filaments. For confocal imaging, cells were cultured on 2- ml of BG-11 solid media containing 1.5% of Gelrite (Fujifilm Wako Pure Chemical Corporation, Japan) instead of agar for clarity, on 35-mm plates with glass bottom (#3910–035, Iwaki, Japan). We confirmed that the colony patterns produced by *Pseudanabaena* under confocal imaging look essentially the same as those observed on agar plates with standard imaging without laser illumination. Macroscopic images of colonies on 90-mm plates (shown in Figs. 1a, 2a, 3a, and 4d,) were taken by a single-lens reflex camera (K-5 II, Pentax, Japan). An LED tracing stand (Arton Smart Tracer Pro A4, Dai-Nippon Bijutu Kougei, Japan) placed underneath the plate was used as a light source (approximately 30 μmol m^− 2^ s^− 1^) for both photography and cell culture. A thermo glass plate (Microwarm Plate KM-1/MP-1000H, Kitazato, Japan) set to 33 °C was placed on top of the agar plates to maintain the growth temperature and avoid condensation. For any microscopic observations except for Fig. S8, cells were culture with illumination from white fluorescent lamps of around 30 μmol m^− 2^ s^− 1^ from the bottom or the upper side to avoid directional effect which may cause phototactic motility as much as possible. For experiment shown in Fig. S8, samples on culture plates were illuminated from lateral direction.

### Image processing and statistical analysis

Images were processed and analyzed with ImageJ 1.50b (NIH, USA) [55] and Fiji 2.0.0-rc-65 [56]. The statistical computing software, R-3.2.3 (64 bit, R Core Team, 2016) [57] was used for statistical analysis.

### Passing account analysis

For passing count images shown in Figs. 2 and 7b, signal density of each image of the time-lapse series was binarized, summed and subjected to logarithmic transformation. Then the result was visualized using a Temporal-Color Code plugin of ImageJ.

### Actual velocity of migrating clusters

The values of actual velocity of each cluster shown in Figs. 3 and 4b was calculated from change in the position of centroid of each cluster on a time-lapse series of tiff images per a frame rate. The values of actual velocity at the peripheral/outmost zone of rotating clusters and that of migrating clusters in Fig. S2 was calculated using positions obtained by manually tracking a certain position of the contour of the disk-like cluster with characteristic shape as a marker, and the tip of the migrating cluster on each time-lapse image, respectively.

### Actual velocity of individual filaments

The values of actual velocity of filaments shown in Fig. 5e was calculated using positions obtained by manually tracking the tip of the filament with ImageJ. The regression line on Fig. 5e is based on the data shown in Fig. S7. We calculated the slope and R-squared value of the linear regression under each length displayed in the horizontal axis of Fig. S7. Although several peaks are shown in Fig. S7b, we chose the dataset under 95.98 μm which was determined to be largest number showing peak of adjusted R-squared to perform linear regression.

### PIV analysis

For PIV analysis shown in Figs. 3 and 4f and d, we employed the PIV plugin of ImageJ [58, 59] to analyze and visualize the cell flow. Flow between two time-lapse images with 10 s interval was calculated with an interrogation window size of 128 px, vector spacing of 64 px and the correlation threshold set of 0.6. Using the resulting data with the PIV plugin, magnitude distribution shown in Fig. 4e was calculated and scaled to the unit of μm/sec. The center position in Fig. 4e was determined by fitting the circle to the disk-like rotating cluster manually.

### Collision angle analysis

Angle of colliding filaments shown in Fig. 5d was manually measured. *Θin* is the incoming angle between the lines with 10-pixel long from each colliding filament to the collision point, while *θout* is the angle between the lines from colliding point to 10-pixel forward to the moving direction of each filament using the line selection tool in Fiji.

### Colony size distribution analysis

The size of any detectable colonies observed in Movie S1 and Movie S15 at indicated time was measured using Fiji. Then, colony size distribution shown in Figs. 2 and 7d–e was analyzed using software package poweRlaw [32] on R-3.2.3 [57], which is based on a method described in Clause et al. [31]. Briefly, we initially fitted the experimental data to the power-law distribution. The estimated power index and the lower cut-off value were set to minimize the Kolmogorov-Smirnov statistic value (*KSd*) of the estimated and the experimental datasets. Then, we performed a goodness-of-fit test. Then, a dataset of composite values was synthesized from the estimated equation. This dataset was prepared so that the ratio of the number of data points above the cut-off value to that below the cut-off value was equivalent to that of the experimental data. For this synthetic dataset, data above the cut-off value were randomly selected to follow the estimated distribution, while data below the cut-off value were randomly sampled from the experimental data below the cut-off value. For each synthetic dataset, the KS value (*KSsim*) was calculated using the same steps as those performed to calculate *KSd*. This process was repeated, following the bootstrapping method, enabling us to determine the probability (*p*) of *KSd* appearing greater than *KSsim*. Clauset et al. [31] have proposed that *H*_*0*_ (the experimental data follows the power-law distribution) should be rejected if the *p*-value is less than 0.1. To select the model distribution, we performed the Vuong’s test, which is a likelihood ratio test for model selection using the Kullback-Leibler criterion [31, 32]. This tests the null hypothesis *H*_*0*_: both distributions are equally far from the true distribution versus the alternative hypothesis *H*_*1*_: one of the test distributions is closer to the true distribution. If the *p*-value given by the test is greater than 0.1, then the test is inconclusive, according to Clauset et al. [31].

### Electron microscopy

Cells were fixed with 2.5% glutaraldehyde dissolved in 0.1 M phosphate buffer solution at 4 °C, washed with phosphate buffer solution, and additionally fixed with osmium tetroxide at 4 °C. Samples were then dehydrated several times in a 50 to 100% ethanol series and then freeze-dried. For SEM observation, dehydrated cells were coated with osmium and observed using a JSM-6320F SEM (JEOL, Japan). For TEM observations, dehydrated cells were embedded in epoxy resin (EPON812; Shell Chemical, USA), and ultrathin slices of the material were doubly stained in uranyl acetate and lead citrate. The sections were coated with carbon in a vacuum vapor deposition system, and then the coated ultrathin slices of cells were observed using TEM (JEM1200EX, JEOL). All observations were performed at the Hanaichi UltraStructure Research Institute (Okazaki, Japan).

### EPS staining assays

For Alcian blue staining, 20 μl of cell suspension was incubated on a slide glass for 30 min. Any floating cells and liquid were removed gently by absorbing to filter paper. The materials remaining on the slide, including the cells, were stained with 20 μl of 1.5% (w/v) Alcian blue 8GX (Sigma-Aldrich, USA) for 30 min. Then, the Alcian blue solution was replaced by 20 μl of BG-11 liquid medium, and a coverslip was then mounted onto the droplet. Stained materials were observed under the microscope (IX-51, Olympus, Japan) with an UplanFLN (10x) objective (Olympus, Japan), and images were collected using the Color CMOS camera (Moticam 5, Motic, China). For ink staining, cells cultured on BG-11 agar medium for about 10 days were used. Twenty microlitre of solution containing 2% india ink (Winsor & Newton, UK), 4μMCaCl_2_, and 0.05% (v/v) Triton X-100 (Sigma) was then dropped on the surface of the medium. Photos were taken under the microscope (IX71) with UPlanFLN (4x) objective (Olympus, Japan) before staining and 2 min after staining.

## Supplementary Information

**Additional file 1: Figure S1.** Phylogenetic analysis based on the 16S RNA gene. 16S rRNA gene sequence of *Pseudanabaena* sp. NIES-4403 was compared to those deposited with the Ribosomal Database Project (RDP), including 24 closely related sequences chosen according to BLAST search results and 5 representative cyanobacterial strains. Numbers on the junctions represent bootstrap values.

**Additional file 2: Figure S2.** Velocity distribution of comet-like wandering clusters and disk-like rotating clusters. The velocity of rotating clusters was measured on the circumference. Stars indicate significancy different distributions with *p*-value of 7.293 × 10^− 9^ with Wilcoxon’s rank sum test.

**Additional file 3: Figure S3.** Trajectories and changes in the velocity and the size of three representative comet-like wandering clusters (video provided with Movie S3).

**Additional file 4: Figure S4.** Negative and positive staining of EPS with india ink and alcian blue, respectively. **a.** Trajectory of comet-like wandering cluster was visualized using india ink. **b.** Extracellular polysaccharide was visualized using alcian blue. Arrowheads indicate the staining of EPS around filaments in some aggregates.

**Additional file 5: Figure S5.** Trajectories and changes in the velocity and the size of three representative disk-like rotating clusters (video provided with Movie S3).

**Additional file 6: Figure S6.** Spatial distribution of the velocity of filaments inside a disk-like rotating cluster by the PIV analysis. Colors indicate the magnitude of PIV (blue, minimal value of 0; red, maximal value of 0.1364566). White circles show standard distance from the center position.

**Additional file 7: Figure S7.** Regression analysis on the filament length and moving velocity. The regression line shown in Fig. 5de is based on the slope (a) and correlation index (adjusted R-squared value) of (b) regression curves when the upper threshold of the filament length is changed. We considered the area from 0 to 100 μm, because this area provided relatively higher correlation indices.

**Additional file 8: Figure S8.** Negative phototactic behavior of *Pseudanabaena*. Cell suspension was put on the center of agar-containing medium on a 90-mm plate and incubated for about 10 days. An arrowhead indicates the direction of illumination.

**Additional file 9: Movie S1.** Time-lapse images of *Pseudanabaena* on a 90-mm agar plate. Cells were inoculated at the center and incubated for about 1 day before recording the video. Colors indicate the position of the cluster shown in Figs. 3 and 4a. For details, see Materials and Methods.

**Additional file 10: Movie S2.** Time-lapse movies of comet-like wandering cluster (extracted from Movie S1).

**Additional file 11: Movie S3.** Time-lapse images on a 90-mm agar plate, which is different from that was shown in Movie S1, with trajectories of wandering clusters shown in Figs. S3a-c. Cells were inoculated at the center and incubated for about 1 day before recording the video.

**Additional file 12**: **Movie S4.** Time-lapse images of wandering or rotating clusters on agar-containing media. A comet-like wandering cluster (upper left) and a disk-like rotating cluster (lower right) are visible. Some bundled filaments and single filaments are also visible.

**Additional file 13: Movie S5.** Time-lapse confocal images of a comet-like wandering cluster.

**Additional file 14: Movie S6**. Time-lapse images of disk-like rotating cluster (extracted from Movie S1).

**Additional file 15: Movie S7.** Time-lapse images on a 90-mm agar plate (same image source for Movie S3) with trajectories of three representative rotating clusters shown in Figs. S5a-c.

**Additional file 16: Movie S8.** Time-lapse confocal images of a disk-like rotating cluster.

**Additional file 17: Movie S9.** Time-lapse images of a bundle composed of aligned filaments. The bundle visible at the beginning of this movie consists of nine filaments.

**Additional file 18: Movie S10.** Time-lapse of images of a single filament following a trail.

**Additional file 19: Movie S11.** Collision between two filaments, moving parallel to each other.

**Additional file 20: Movie S12.** Collision between two comet-like clusters, forming a unified cluster.

**Additional file 21: Movie S13.** Transition from a comet-like cluster to a rotating cluster.

**Additional file 22**: **Movie S14.** Collision between a comet-like cluster and a disk-like rotating cluster, leading to the collapse of the rotating cluster.

**Additional file 23: Movie S15.** Video of the simulated result of the self-propelled particle model.

**Additional file 24: Movie S16.** Time-lapse images of a representative wandering cluster on simulation (extracted from Movie S15). Color indicates the trajectory of cluster from cyan (at 1000 steps) to magenta (at 2000 steps).

**Additional file 25: Movie S17.** Time-lapse images of a representative rotating cluster on simulation (extracted from Movie S15). Color indicates the trajectory of cluster from cyan (at 1000 steps) to magenta (at 2000 steps).

**Additional file 26: Dataset S1.** Original dataset used for figures and tables were compiled.

## Data Availability

All the datasets used are available as the Dataset SI file. The strain used in the current study is available at https://mcc.nies.go.jp/strainList.do?strainId=4401 from the Microbial Culture Collection at the National Institute for Environmental Studies (NIES collection, Japan) or the corresponding author on reasonable request. Source code for simulation is available at https://github.com/yh1984/Hisamoto_Yamamoto2020.

## Abbreviations

CDF: Cumulative distribution function
EPS: Extracellular polysaccharides
JPC: Junctional pore complex
KS: Kolmogorov-Smirnov (value, statistic)
PDF: Probability distribution function
PIV: Particle image velocimetry
