# Supplementary figures and images for: Scattered migrating colony formation in the filamentous cyanobacterium, Pseudanabaena sp. NIES-4403
Source: BMC Microbiol. 2021 Aug 16;21:227. doi: 10.1186/s12866-021-02183-5 (PMC8365994; doi:10.1186/s12866-021-02183-5)

Figure S1

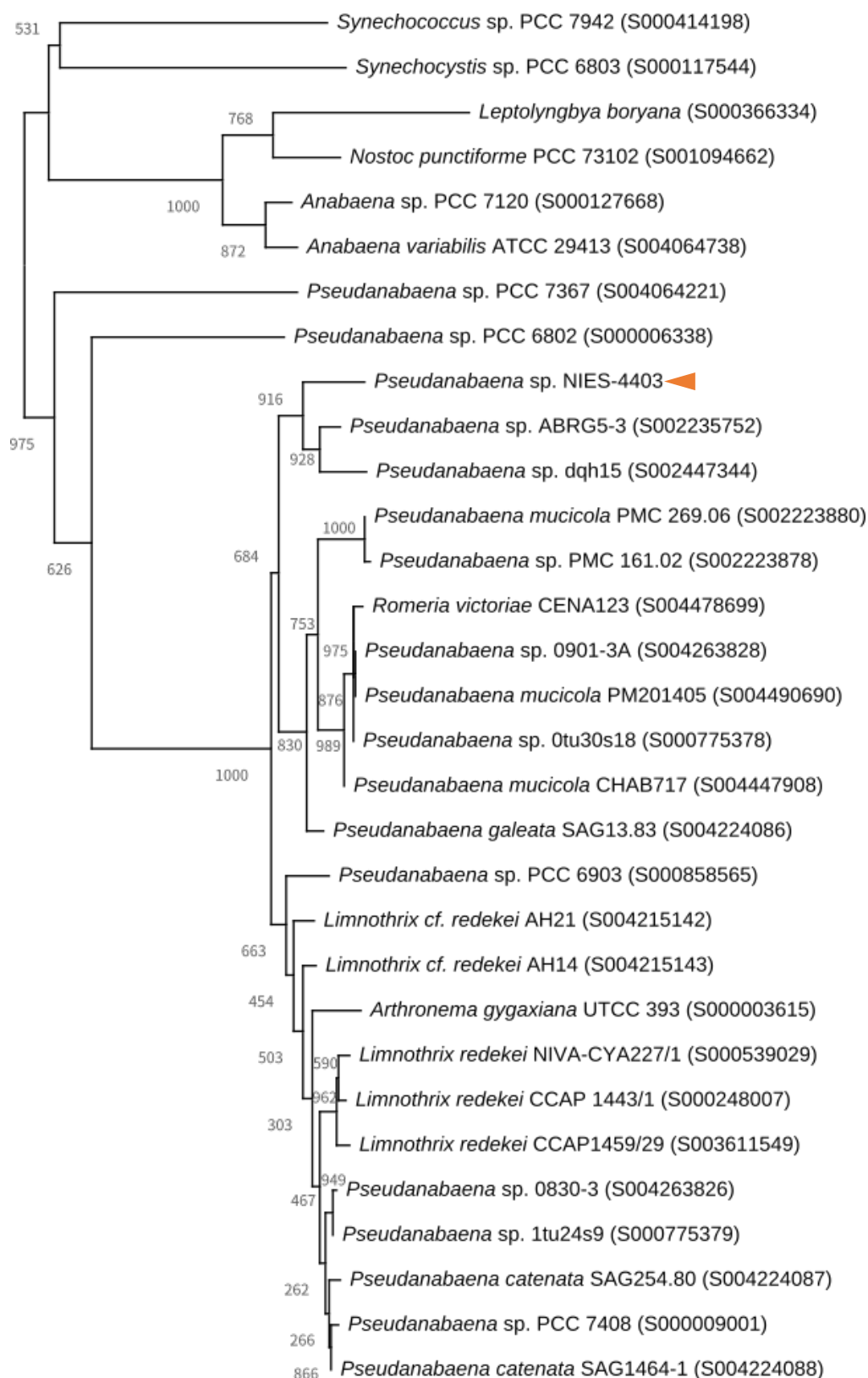

1000

0.020

Supplement: Supplementary file 1 — Additional file 1: Figure S1. Phylogenetic analysis based on the 16S RNA gene. 16S rRNA gene sequence of Pseudanabaena sp. NIES-4403 was compared to those deposited with the Ribosomal Database Project (RDP), including 24 closely related sequences chosen according to BLAST search results and 5 representative cyanobacterial strains. Numbers on the junctions represent bootstrap values. [file 12866_2021_2183_MOESM1_ESM.pdf]

Figure S2

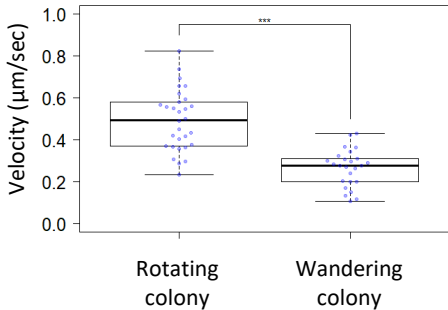

Supplement: Supplementary file 2 — Additional file 2: Figure S2. Velocity distribution of comet-like wandering clusters and disk-like rotating clusters. The velocity of rotating clusters was measured on the circumference. Stars indicate significancy different distributions with p-value of 7.293 × 10− 9 with Wilcoxon’s rank sum test. [file 12866_2021_2183_MOESM2_ESM.pdf]

Figure S3

**a**

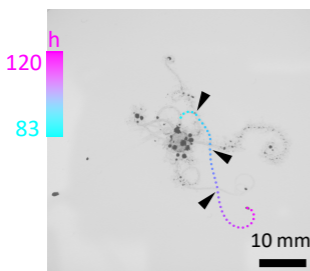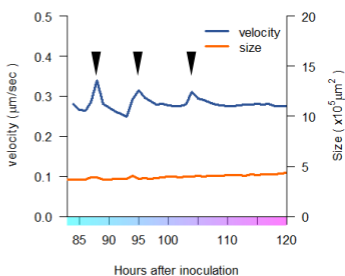

**b**

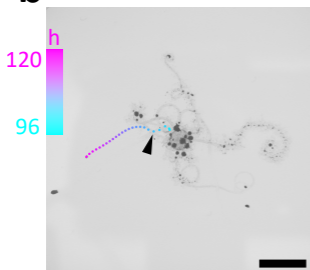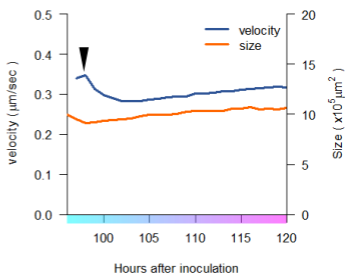

**c**

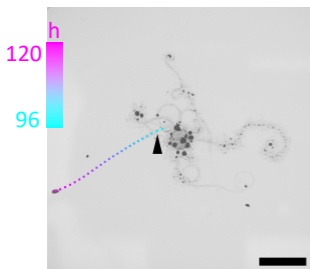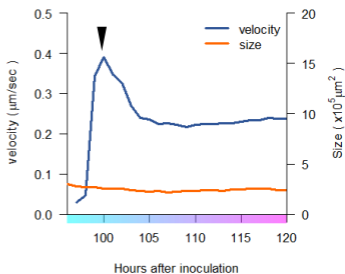

Supplement: Supplementary file 3 — Additional file 3: Figure S3. Trajectories and changes in the velocity and the size of three representative comet-like wandering clusters (video provided with Movie S3). [file 12866_2021_2183_MOESM3_ESM.pdf]

Figure S4

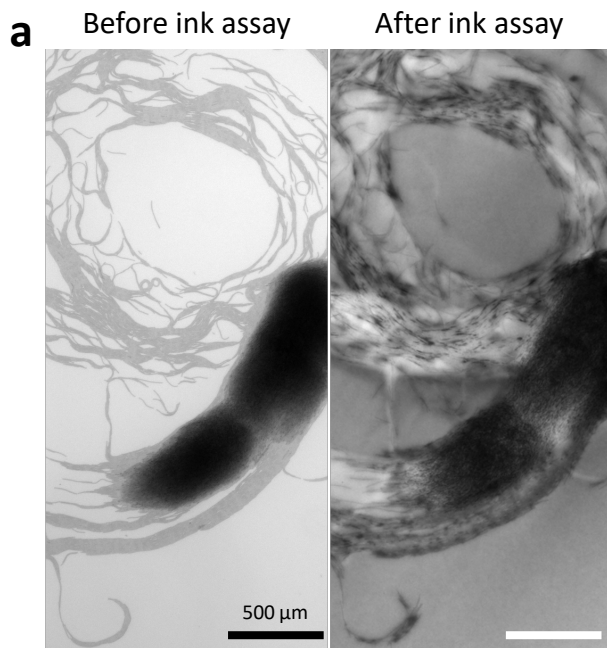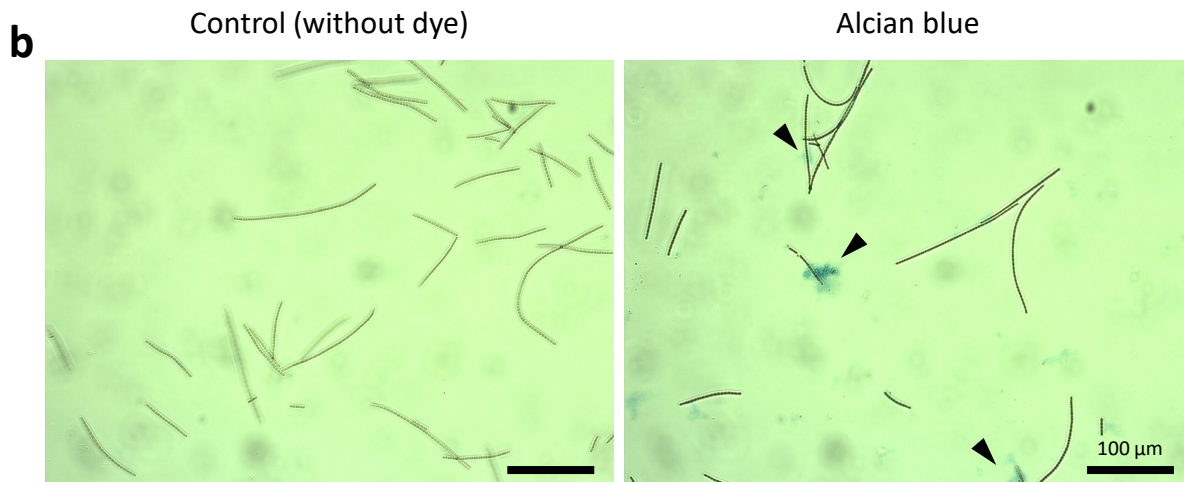

Supplement: Supplementary file 4 — Additional file 4: Figure S4. Negative and positive staining of EPS with india ink and alcian blue, respectively. a. Trajectory of comet-like wandering cluster was visualized using india ink. b. Extracellular polysaccharide was visualized using alcian blue. Arrowheads indicate the staining of EPS around filaments in some aggregates. [file 12866_2021_2183_MOESM4_ESM.pdf]

Figure S5

**a**

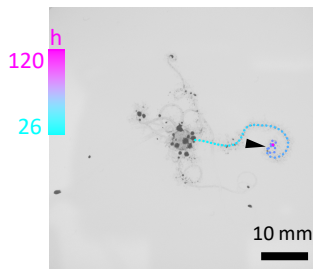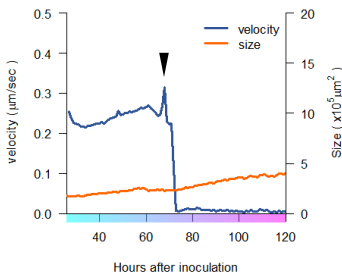

**b**

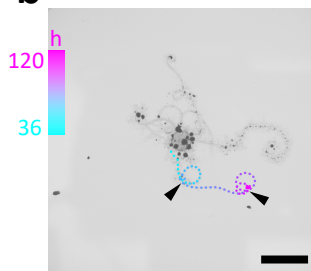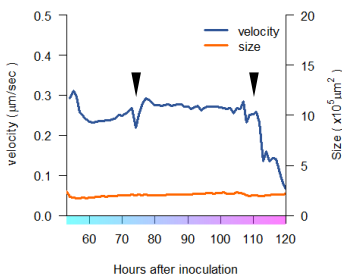

**c**

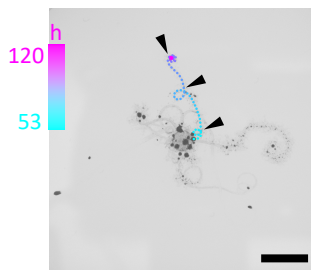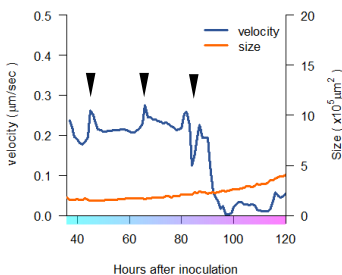

Supplement: Supplementary file 5 — Additional file 5: Figure S5. Trajectories and changes in the velocity and the size of three representative disk-like rotating clusters (video provided with Movie S3). [file 12866_2021_2183_MOESM5_ESM.pdf]

Figure S6

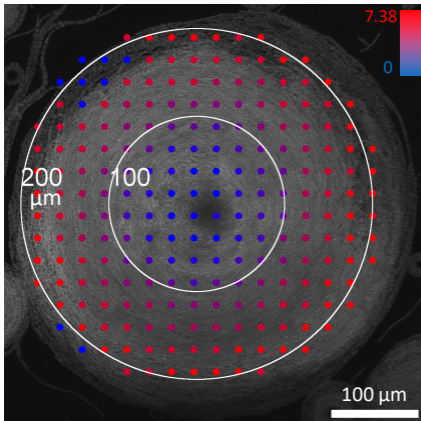

Supplement: Supplementary file 6 — Additional file 6: Figure S6. Spatial distribution of the velocity of filaments inside a disk-like rotating cluster by the PIV analysis. Colors indicate the magnitude of PIV (blue, minimal value of 0; red, maximal value of 0.1364566). White circles show standard distance from the center position. [file 12866_2021_2183_MOESM6_ESM.pdf]

Figure S7

**a**

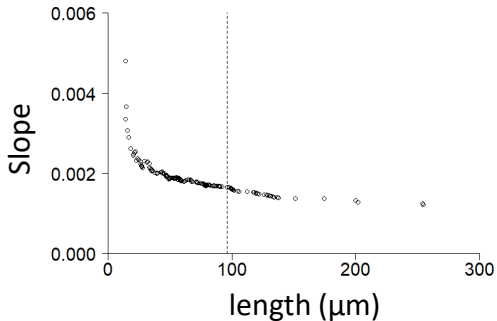

**b**

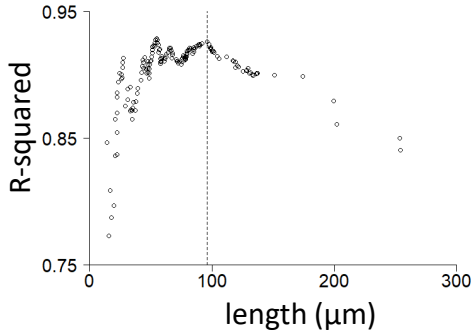

Supplement: Supplementary file 7 — Additional file 7: Figure S7. Regression analysis on the filament length and moving velocity. The regression line shown in Fig. 5de is based on the slope (a) and correlation index (adjusted R-squared value) of (b) regression curves when the upper threshold of the filament length is changed. We considered the area from 0 to 100 μm, because this area provided relatively higher correlation indices. [file 12866_2021_2183_MOESM7_ESM.pdf]

Figure S8

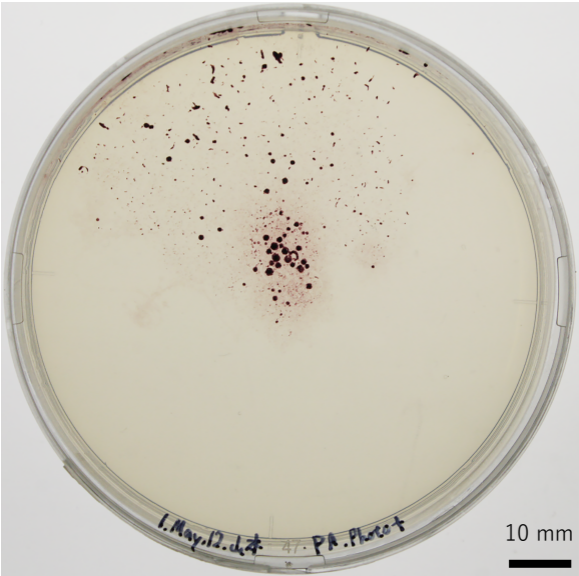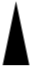

Supplement: Supplementary file 8 — Additional file 8: Figure S8. Negative phototactic behavior of Pseudanabaena. Cell suspension was put on the center of agar-containing medium on a 90-mm plate and incubated for about 10 days. An arrowhead indicates the direction of illumination. [file 12866_2021_2183_MOESM8_ESM.pdf]
